# Thirty Years of Experience with High-Voltage Injuries: Mechanisms, Current Flow Patterns, and Implications for Cardiac and Renal Failure in Train-Surfing vs. Work-Related Cases

**DOI:** 10.3390/jcm14082659

**Published:** 2025-04-13

**Authors:** Viktoria Koenig, Julian Joestl, Gerald Ihra, Marita Windpassinger, Maximilian Monai, Alexandra Fochtmann-Frana

**Affiliations:** 1 Division of Plastic, Aesthetic and Reconstructive Surgery, Medical University of Vienna, Waehringer Guertel 18–20, A-1090 Vienna, Austria; n11927646@students.meduniwien.ac.at (M.M.); alexandra.fochtmann@meduniwien.ac.at (A.F.-F.); 2 Private Clinic, Spitalgasse 19, A-1090 Vienna, Austria; 3 Division of General Anaesthesia and Intensive Care Medicine, Medical University of Vienna, Waehringer Guertel 18–20, A-1090 Vienna, Austria; gerald.ihra@meduniwien.ac.at (G.I.); marita.windpassinger@meduniwien.ac.at (M.W.)

**Keywords:** high-voltage electrical injuries, train surfing, cardiac failure, arrhythmia, kidney dysfunction, current flow mechanisms

## Abstract

**Background:** High-voltage injuries pose severe risks, particularly among train surfers and occupational workers. This study compares current flow mechanisms and their association with cardiac failure and kidney function problems in these groups. **Methods**: A retrospective analysis of 102 patients treated for high-voltage injuries between 1994 and 2024 was conducted, including 32 train-surfing and 70 work-related cases. Demographics, current flow patterns, and complications were analyzed. **Results**: Train surfers, mostly young males (median age: 19 years), sustained more severe burns compared to older males with work-related injuries (median age: 34 years), with a %TBSA of 47.6% vs. 25.4% (*p* < 0.0001). Vertical electrical flow was predominant among train surfers (65.62%) and led to cardiac failure in 37.5% of cases. In contrast, diagonal flow was most common in work-related injuries (58.57%). Cardiac failure occurred in 21.57% of all patients, with vertical flow posing the highest risk (38.46%). Kidney failure affected 43.75% of train surfers and 21.43% of work-related cases, with dialysis required in 21.57% overall. Train surfers were more likely to require resuscitation (43.75% vs. 15.71%, *p* = 0.005), while ECG findings showed no significant differences (*p* = 0.325). Biomarker levels, such as CK, myoglobin, and troponin, were significantly higher in train surfers, reflecting greater muscle damage. **Conclusions**: Current flow mechanisms significantly influence cardiac and renal complications. Vertical flow, common in train surfers, is strongly associated with cardiac failure, while work-related injuries exhibit varied flow patterns. These findings highlight the need for targeted prevention strategies and public awareness.

## 1. Introduction

High-voltage electrical injuries pose significant challenges in trauma and reconstructive surgery due to their extensive impact on multiple organ systems [1,2,3,4]. These injuries are common in work-related settings, where high-voltage exposure frequently leads to severe burns, amputations, and systemic complications such as cardiac arrhythmias and kidney failure [5,6]. Apart from these work-related accidents, Train-surfing injuries are a devastating subset of electrical trauma arising from the hazardous practice of climbing and riding on the outside of moving trains [6,7,8,9]. This high-risk behavior frequently exposes individuals, often young thrill-seekers, to high-voltage currents from overhead power lines or electrified train systems [10,11,12,13]. The absence of safety measures and the unpredictability of these incidents result in severe and often fatal injuries, including extensive burns, amputations, and life-threatening complications [3,8,14,15,16]. Unlike occupational injuries, these incidents lack regulated safety frameworks, making them particularly challenging to address [9,13,17].

The severity of electrical injuries, including those sustained by train surfers, is also determined by the pathway of current through the body [17,18]. Electrical energy follows a path of least resistance, with conductive tissues like blood vessels and nerves sustaining less damage than high-resistance tissues such as bone and fat, which absorb energy and suffer localized thermal injury [17]. True electrical burns, where the body becomes part of the circuit, are particularly severe, marked by entrance and exit wounds and extensive tissue damage proportional to current intensity [2,13,15,19].

Electrical injuries are categorized as high-voltage (>1000 volts) or low-voltage (<1000 volts) [14,18]. High-voltage injuries, common in railway and industrial settings, often cause deep tissue destruction due to direct current flow, while low-voltage injuries, typically domestic, result in superficial burns [17]. Electric arcs, generating temperatures up to 5000 °C, can cause severe thermal burns even without direct contact [19]. Skin resistance, influenced by factors like moisture or damage, determines whether current penetrates deeper tissues, with high-voltage energy often leading to internal burns [20]. Alternating current (AC), prevalent in railway systems, is three times more destructive than direct current (DC) at equivalent voltages due to prolonged muscle contractions that exacerbate exposure [8,12,17].

Electrical injuries range from minor burns to life-threatening internal damage, with the heart being a critical organ at risk [21,22]. Although commonly encountered in both domestic and occupational settings, clinical management remains challenging due to limited literature and inconsistent guidelines. The risk of delayed cardiac arrhythmias is a key concern, especially in high-voltage injuries, and may warrant extended monitoring in selected cases [23,24]. Apart from the path of current flow, several factors contribute to the risk of cardiac failure in high-voltage injuries [24,25]. Voltage plays a key role, as higher voltages increase tissue damage, current penetration, and the likelihood of arrhythmias [24]. Other critical factors include current intensity, exposure duration, frequency (with AC being more arrhythmogenic than DC), and individual susceptibility, such as pre-existing heart conditions or electrolyte imbalances [17,20,26].

Understanding the direction of electrical current and its physiological impact is essential for reducing complications and improving outcomes in electrical trauma. This study explores the distinct current flow patterns and injury mechanisms observed in occupational high-voltage incidents versus train-surfing accidents, offering deeper insight into the underlying pathophysiology and informing more effective prevention strategies.

Despite the clinical relevance, the existing literature rarely differentiates between low- and high-voltage injuries or investigates the specific complications associated with extreme high-voltage trauma, such as that seen in train surfing. Although electrical injuries are frequently encountered in emergency settings, there are currently no established guidelines or consensus recommendations for their management in Europe. Reports of delayed arrhythmias are rare, and their direct association with electrical trauma remains uncertain. While cardio-specific biomarkers are often used to assess the risk of myocardial injury, robust evidence supporting their clinical utility in this context is lacking [22]. This study addresses that gap by presenting long-term, comparative data on high-voltage injuries from both contexts, with a particular focus on identifying predictors of cardiac and renal complications, the need for resuscitation, and mortality. The findings emphasize the critical role of current flow direction and injury mechanism in shaping clinical outcomes and highlight the need for tailored prevention and management approaches.

## 2. Materials and Methods

After obtaining ethical board approval (Ethics Committee Approval No. 1384/2023), a retrospective review of patient records was conducted for individuals admitted between January 1994 and December 2024 with high-voltage injuries related to train surfing or work-related circumstances. In our patient cohort, accidents predominantly occurred on overhead high-voltage lines of the national railway system, which typically carry approximately 15,000 volts. Therefore, it is reasonable to assume this voltage level in the affected patients. The actual severity of the injury, however, depends on multiple influencing factors—such as current type, amperage, voltage, resistance, duration of exposure, and environmental conditions like humidity or temperature. These variables, often referred to as Kouwenhoven’s factors, significantly affect tissue damage but are difficult to reconstruct retrospectively and could not be assessed in detail in our study population. Data were retrieved from the hospital’s electronic medical record system.

Patients with documented high-voltage electrical injuries (≥1000 V) associated with train surfing or work-related accidents were included in the study. Exclusion criteria were low-voltage injuries (<1000 V) and cases treated outside the department. The parameters analyzed included age, sex, injury circumstances, total burn surface area (%TBSA), type of current flow through the body, cardiac failure, ECG findings, renal failure, and dialysis requirements. No age or gender restrictions were applied, ensuring that all individuals affected by these uncommon injury patterns were considered. Patients with low-voltage injuries (<1000 V) or those treated outside the department were excluded. The final study population consisted of 102 patients aged between 13 and 59 years, including 5 females.

Treatment protocols initially followed a standardized approach, focusing on fluid resuscitation with modifications guided by urinary output, hematocrit, and serum lactate levels. Myoglobinuria was addressed with diuretics to maintain an appropriate urinary pH, supported by central venous access and continuous hemodynamic and respiratory monitoring. With the adoption of updated standards, treatment has evolved into a more sophisticated and personalized strategy. Fluid resuscitation incorporates balanced solutions, including crystalloids, albumin, and high-dose vitamin C, administered in the lowest effective volumes. Therapy is directed by advanced monitoring tools such as pulse contour cardiac output (PiCCO), hemoglobin levels, and echocardiographic evaluations. Circulatory support includes catecholamine administration (e.g., noradrenaline, dobutamine), supplemented by comprehensive ICU management, including oxygen therapy, temperature regulation, and effective pain control to ensure optimal and individualized care.

The initial diagnostic evaluation included consultations in ENT and ophthalmology, alongside imaging studies such as ultrasound and CT scans for stabilized patients. Laboratory monitoring included regular assessments of creatine kinase and myoglobin levels, performed every 6 h in the first 24 h and then daily thereafter. Wounds were disinfected and dressed daily, and severe burns were managed with skin grafts or free flaps. Preoperative CT angiography was used to plan microsurgical reconstructions.

Cardiac failure was identified based on clinical findings, including hemodynamic instability, ECG changes indicative of arrhythmias or myocardial injury, and elevated cardiac biomarkers (e.g., troponin). This definition includes both electrical findings and associated clinical symptoms suggestive of functional cardiac compromise following high-voltage injury. Renal failure was defined according to changes in serum creatinine levels, urine output, and, where available, markers of acute kidney injury (AKI). These definitions were applied consistently across patient groups to allow for accurate comparisons.

Current flow patterns were classified as vertical, diagonal, or horizontal based on documented entry and exit points. The vertical flow was defined as a cranial or upper-body entry with a caudal exit (e.g., hand to foot), diagonal flow as oblique cross-body conduction (e.g., right hand to left foot), and horizontal flow as same-level conduction (e.g., hand to hand). Cases with unclear documentation were classified as undefined.

### 2.1. Statistics

Statistical tests were performed using R Version 3.1.1 SPSS (Armonk, NY, USA). Continuous variables were analyzed using the Student’s *t*-test for parametric data and the Mann–Whitney U test for non-parametric data. Categorical variables were assessed using the chi-square test or Fisher’s exact test, where appropriate. Correlations between current flow type and complications such as cardiac failure and renal dysfunction were evaluated using contingency tables and chi-square tests. Results were considered statistically significant at *p* < 0.05. Parametric data are reported as mean ± standard deviation, while non-parametric data are presented as median (minimum–maximum).

To identify potential predictors of clinical outcomes, three separate multivariate logistic regression analyses were performed with the following binary outcomes: mortality, acute kidney failure, and cardiac failure. Independent variables included demographic factors, clinical parameters (including the presence and type of ECG abnormalities), and injury characteristics. Variables were selected based on clinical relevance and data availability.

### 2.2. Source of Funding

This study did not receive any external funding.

### 2.3. Declaration of Generative AI and AI-Assisted Technologies

During the preparation process of this work, ChatGPT (version: GPT-4) was used in order to visualize the analyzed data. After using this tool, the authors reviewed and edited the content as needed and take full responsibility for the content of the publication.

## 3. Results

One hundred and two patients were treated for high-voltage injuries between 1994 and 2024, including 32 train-surfing and 70 work-related injuries. Train surfers were predominantly young males, with a median age of 19 years (range: 13–36 years), while work-related injuries involved a median age of 34 years (range: 18–59 years). The extent of burns was significantly higher in train-surfing patients, with a mean TBSA (total body surface area) of 47.6% (±20.1%), compared to 25.4% (±17.8%) in work-related cases (*p* < 0.001).

In all patients, 36.27% had associated traumatic injuries, with a higher prevalence in train-surfing patients (56.25%) compared to work-related patients (27.14%) (*p* = 0.008). The most common injuries included intracerebral bleeding (ICB), vertebral fractures, or spinal cord injuries, with several patients sustaining multiple traumata.

A total of 22 patients (21.57%) experienced cardiac failure, with a significantly higher prevalence among train surfers (37.5%) compared to work-related injuries (14.29%) (*p* = 0.015) (Table 1).

Regarding ECG findings, 55.88% of all patients exhibited abnormalities, with train surfers showing a higher prevalence (62.5%) compared to work-related cases (52.86%), though this difference was not statistically significant (*p* = 0.274).

A chi-square test revealed a statistically significant association between current flow type and cardiac failure (chi-square statistic = 8.31; *p* = 0.040; significant at *p* < 0.05). Vertical current flow accounted for the highest proportion of cardiac failure cases (38.46%), followed by diagonal flow (15.91%). No cardiac failure was observed with horizontal flow. Among diagonal flow subtypes, cardiac failure was more common when the right hand was the entry point (57.14%) compared to the left hand (42.86%).

Current flow patterns varied significantly between train surfers and work-related cases (*p* < 0.001). Current flow patterns were classified as vertical, horizontal, or diagonal, depending on the presumed trajectory of the electrical current through the body. Vertical current flow refers to pathways such as from head to foot, horizontal flow typically describes current passing from hand to hand or across the chest, and diagonal flow includes trajectories from an upper extremity to the contralateral lower extremity (Table 2).

**Figure 1:**
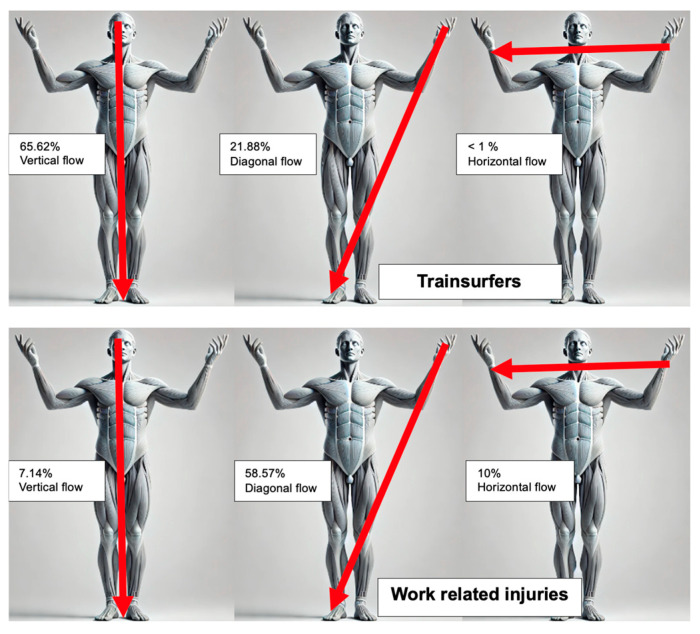
Current flow in train surfers and work-related injuries.

Train-surfing injuries predominantly involved vertical current flow (65.62%), often entering through the head or upper extremities and exiting at the lower extremities or arm. Secondary trauma, including brain damage, cranial bleeding, and fractures, frequently occurred due to falls following electrical arcs. Work-related injuries, on the other hand, predominantly exhibited diagonal current flow (58.57%), reflecting the use of tools and hands during occupational tasks. Horizontal flow was observed in 10.00% of cases, while vertical flow accounted for 7.14%. Entry points for work-related injuries were commonly in the upper extremities, particularly the hands, with exit points in the lower extremities (Figure 1).

Among all patients, 113 ECG findings occurred. Sinus tachycardia was the most common finding, observed in 18 cases. Ventricular extrasystoles, atrial fibrillation, and sinus bradycardia each occurred in six cases. Other notable findings included ventricular fibrillation in five cases and supraventricular tachycardia in three cases. Less frequent findings included ST elevation, sinus tachycardia with right bundle branch block (RBBB), and sinus bradycardia with RBBB, each observed in two cases. Rare findings, each appearing in one case, were ventricular tachycardia, negative T waves, negative T waves with RBBB, atrial fibrillation with ST elevation, and bigeminus with RBBB (Table 3).

Among all resuscitated patients, the most frequent ECG finding was sinus tachycardia, observed in seven cases. Ventricular fibrillation followed, occurring in four patients, while sinus bradycardia was recorded in three. Two patients showed no abnormalities, and another two presented with atrial fibrillation. Additional findings included sinus tachycardia combined with right bundle branch block (RBBB) in two cases, while ventricular tachycardia, negative T waves (with and without RBBB), ventricular extrasystoles, and ST elevation were each seen in one patient.

**Figure 2:**
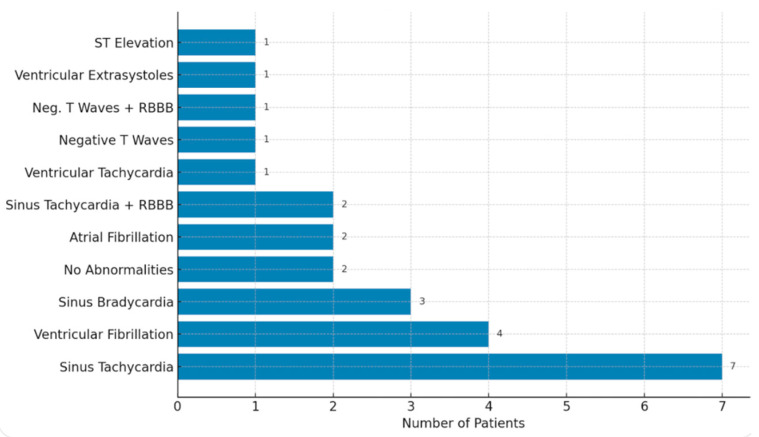
ECG findings among resuscitated patients.

When analyzing resuscitation rates, 43.75% of train surfers required resuscitation, while only 15.71% of work-related cases needed resuscitation. This difference was statistically significant (*p* = 0.005). For train surfers requiring resuscitation, sinus tachycardia, and sinus bradycardia were each identified in two cases. Two patients had no abnormalities, while atrial fibrillation, ventricular fibrillation, sinus tachycardia with RBBB, ventricular tachycardia, negative T waves (both with and without RBBB), and ventricular extrasystoles were each noted in one case. No cases of ST elevation were observed in train surfers. In work-related cases requiring resuscitation, sinus tachycardia was the predominant finding, present in five patients. Ventricular fibrillation appeared in three cases, while sinus bradycardia, atrial fibrillation, and ST elevation were each seen in one patient. No abnormalities, sinus tachycardia with RSB, ventricular tachycardia, negative T waves, or ventricular extrasystoles were observed in this group (Figure 2).

Overall, sinus tachycardia was the most common finding across all groups, particularly in work-related injuries. Train surfers demonstrated a wider variety of ECG abnormalities, reflecting the diverse cardiac impacts of their injuries. Ventricular fibrillation and ST elevation were more prominent in work-related cases, suggesting more severe cardiac complications in these situations.

Statistical analysis revealed a significant difference in resuscitation rates between train surfers and work-related cases (*p* = 0.005), with train surfers being more likely to require resuscitation (43.75% vs. 15.71%). However, no significant difference was found in the distribution of ECG findings between the two groups (*p* = 0.325), indicating that the observed variations in cardiac abnormalities could be due to chance. These results highlight the greater severity of injuries among train surfers while suggesting no substantial differences in the types of ECG abnormalities observed.

Logistic regression analysis confirmed that both cardiac failure (*p* = 0.008) and resuscitation (required in 28 patients; 27.5% overall, with 14 of 32 train surfers; 43.8% vs. 11 of 70 work-related cases; 15.7%, *p* = 0.005) were strong, independent predictors of mortality (mortality rate: 15.7% overall).

These findings are consistent with the clinical presentation of train-surfing injuries, which are typically more severe—characterized by higher total body surface area (TBSA) burns and vertical current flow—factors that likely contribute to the increased risk of life-threatening cardiac complications in this patient group.

To further investigate the prognostic relevance of specific ECG abnormalities, a logistic regression analysis was performed using mortality as the outcome variable. Among the various ECG patterns, ventricular fibrillation emerged as the only significant independent predictor of mortality (OR 3.42, 95% CI 0.75–6.09, *p* = 0.012). Other ECG findings, such as sinus tachycardia, bradycardia, ST elevation, or atrial fibrillation, did not show a statistically significant association with death. This suggests that while a broad range of rhythm disturbances may be observed following high-voltage injury, ventricular fibrillation specifically identifies patients at markedly increased risk. These results highlight the importance of immediate rhythm assessment and aggressive intervention in this subgroup to improve outcomes (Table 4).

**Figure 3:**
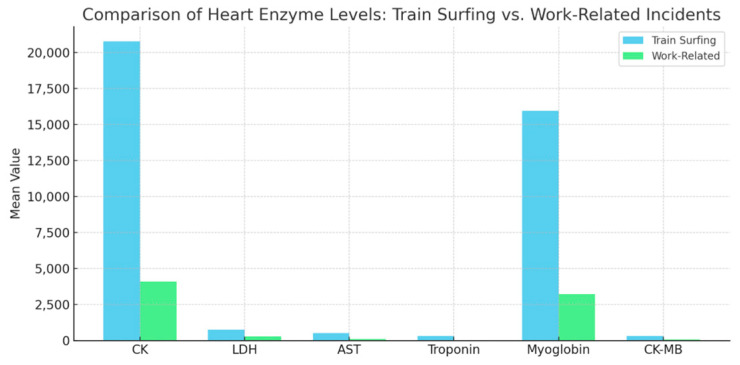
Cardiac biomarkers in train surfers and work-related injuries.

Train-surfing incidents result in significantly higher mean levels of biomarkers compared to work-related injuries, reflecting the severe impact of high-voltage trauma. For instance, creatine kinase (CK) levels (U/L) average 20,778.73 in train surfers compared to 4097.50 in work-related cases (*p* < 0.001), while myoglobin levels (ng/mL) average 15,951.83 versus 3232.10, respectively, (*p* < 0.001). These disparities highlight the extent of muscle damage and rapid breakdown in train-surfing injuries, further emphasized by the pronounced differences in cardiac-specific markers like troponin (ng/L), which averages 309.08 ng/L in train surfers compared to 27.91 ng/L in work-related incidents (*p* = 0.002) (Figure 3).

Kidney failure was identified in 29 patients (28.43%). Among these, 43.75% of train surfers and 21.43% of work-related cases were affected (*p* = 0.031). Dialysis was required in 22 patients (21.57%), with a higher proportion among train surfers (34.38%) compared to work-related cases (15.71%) (*p* = 0.022). However, no significant correlation was found between current flow type and kidney failure (chi-square statistic = 3.84; *p* = 0.279; not significant).

To explore the predictors of organ complications in high-voltage injuries, two separate multivariate logistic regression analyses were conducted for cardiac failure and acute kidney failure. For cardiac failure, none of the examined variables reached statistical significance. Although train surfing was associated with a higher likelihood of cardiac complications (*p* = 0.158), this trend did not achieve statistical significance. Age at the time of the accident (*p* = 0.968), ECG abnormalities (*p* = 0.997), and specific entry or exit points—such as entry via the head (*p* = 0.302), upper extremities (right: *p* = 0.998; left: *p* = 0.993), or exit through the lower extremities (right: *p* = 0.267; left: *p* = 0.997), arm (*p* = 0.993), or double lower limb exit (*p* = 0.991) were not independently predictive of cardiac failure in this model. The lack of statistical significance across predictors may reflect the limited number of cardiac failure events and the presence of multicollinearity or separation in the dataset (Table 5).

In contrast, the model for acute kidney failure yielded one significant predictor. The presence of ECG abnormalities was independently associated with renal complications (*p* = 0.007), suggesting a link between myocardial strain or systemic muscle damage and renal impairment. Train surfing, again, demonstrated a non-significant trend toward increased risk (*p* = 0.151), while other factors such as age (*p* = 0.860), entry via head (*p* = 0.681), upper extremities (right: *p* = 0.937; left: *p* = 0.772), and exit sites (right leg: *p* = 0.790; left leg: *p* = 0.956; arm: *p* = 0.934; double lower limb: *p* = 0.930) showed no independent effect on renal outcome (Table 6).

Taken together, these results highlight ECG abnormalities as a meaningful clinical indicator of renal involvement in high-voltage injuries, whereas cardiac failure appears to result from a more complex interplay of factors not fully captured by this model. The findings support further investigation into organ-specific injury mechanisms and underline the importance of individualized risk assessment in this heterogeneous patient group.

A comparison of mortality rates between work-related and train-surfing high-voltage injuries revealed a noticeable difference. Among the 70 occupational cases, eight patients died, corresponding to a mortality rate of 11.43%. In contrast, the train-surfing group experienced the same number of deaths (*n* = 8) out of only 32 cases, resulting in a significantly higher mortality rate of 25.0%. Despite this numerical disparity, the difference was not statistically significant (*p* = 0.1456).

To further explore potential risk factors for mortality, a multivariate logistic regression was conducted on the full cohort of 102 patients. After adjusting for confounding variables, three factors emerged as independent predictors of death: total body surface area burned (TBSA) (OR 1.08, 95% CI 1.02–1.15, *p* = 0.009), the need for resuscitation (OR 4.56, 95% CI 1.11–18.79, *p* = 0.035), and the presence of acute kidney failure (OR 3.27, 95% CI 1.09–9.76, *p* = 0.034). Belonging to the train-surfing group was associated with a trend toward increased mortality risk, though this did not reach statistical significance (OR 2.11, 95% CI 0.79–5.63, *p* = 0.129).

## 4. Discussion

The review by Waldmann et al. highlights the diagnostic and therapeutic uncertainty that often surrounds the management of electrical injuries, particularly due to the lack of standardized guidelines and the unpredictable nature of internal organ damage [24]. Their emphasis on the need for early risk stratification and careful cardiac monitoring is well reflected in our findings, where 55.9% of patients exhibited ECG abnormalities, and 21.6% developed clinically significant cardiac failure. Unlike low-voltage cases, which may be safely discharged under defined conditions as Waldmann et al. suggest, our data show that high-voltage injuries—especially in train-surfing patients—are associated with a significantly higher incidence of arrhythmias, resuscitation, and biomarker elevation, justifying intensive surveillance. These observations support the call for tailored management strategies and underscore the need for public and occupational safety education to prevent such high-risk injuries.

However, a large Danish cohort study found no significant increase in long-term cardiac morbidity or mortality among patients after electric shock compared to the general population, suggesting that routine prolonged observation may not be necessary for all [23]. Still, case reports highlight that myocardial injury can occur even in non-lethal shocks, reinforcing the need for individualized risk assessment, early cardiac evaluation (e.g., ECG, biomarkers, imaging), and improved public and occupational safety measures. This summary underscores the importance of a balanced approach—vigilant initial assessment without unnecessary over-monitoring in low-risk cases.

The comparison of train-surfing injuries and work-related high-voltage injuries highlights distinct patterns in demographics, injury mechanisms, and associated complications. Our study identified train surfers as predominantly young males with a median age of 19 years, consistent with findings by Lumenta et al., who also reported a significantly younger population among train surfers (mean age: 15.8 years) compared to other high-voltage injury groups [13]. Similarly, Koller reported a predominance of adolescent males involved in high-tension injuries from railway overhead cables, further emphasizing the unique demographic associated with train surfing [12].

The mean total burn surface area (TBSA) of 47.6% among train surfers in our study was higher than the 25.4% in work-related injuries, aligning with Lumenta et al.’s observation of a mean TBSA of 49.7% among train surfers [13]. This suggests that contact with overhead power lines typically results in more extensive burns due to the direct and prolonged exposure to electrical arcs. By contrast, Hussmann et al. found a lower mean TBSA of 9.5% across all high-voltage injuries, likely due to a broader inclusion of less severe cases or different injury patterns of the patients [7].

Vertical current flow, observed in 65.62% of train-surfing injuries, was a key contributor to cardiac failure in our study, with 37.5% of train surfers experiencing this complication. Lumenta et al. similarly noted a high incidence of cardiac injuries among train surfers, attributed to the nature of the vertical current passing through vital structures such as the heart [13]. In contrast, diagonal current flow, predominant in work-related injuries (58.57%), reflects the frequent use of tools and hand contact with electrical sources during occupational tasks. These findings align with those of Zhu et al., who highlighted diagonal current flow as a common pattern in occupational settings due to tool-mediated injuries [27].

Our findings do not completely align with those of Ferreira et al., as we also found no connection between the current pathway and renal failure; however, we observed significant differences in relation to cardiac failure, ECG findings, and cardiac biomarkers [28]. While Ferreira et al. reported no association with cardiac arrhythmia, our results suggest a potential link that warrants further investigation.

Regarding renal failure, our study reported a prevalence of 28.43%, with a higher proportion among train surfers (43.75%) compared to work-related cases (21.43%). While we specifically analyzed the relationship between current flow type and cardiac or renal complications, it is important to note that renal failure is commonly linked to extensive muscle damage and systemic release of myoglobin, a well-documented sequela of high-voltage injuries [9,16,29,30]. Train surfers, due to their higher TBSA and prolonged exposure to electrical arcs, are particularly susceptible to this mechanism. Similar observations were reported by Hussmann et al., who highlighted the role of extensive muscle necrosis and subsequent myoglobinuria as key contributors to renal dysfunction in high-tension injuries [7].

ECG abnormalities were more prevalent in train surfers (62.5%) compared to work-related cases (52.86%), with sinus tachycardia being the most common finding. This pattern is consistent with the high proportion of cardiac complications observed in high-voltage injuries [8,28]. Hussmann et al. similarly identified cardiac arrhythmias in a substantial proportion of high-tension cases, underscoring the vulnerability of the cardiovascular system to electrical injuries [7].

The high incidence of ventricular fibrillation (VF) and supraventricular tachycardia (SVT) in our study aligns with previous findings by Guinard et al., who reported a high prevalence of persistent cardiac abnormalities following high-voltage electrocution [31]. Our data also show that VF was more common in work-related injuries. However, it contrasts with Hansen et al., who found that most patients did not develop severe cardiac complications following electrical injuries [23]. Interestingly, our results did not show a significant difference in ECG abnormality distribution between train surfers and work-related cases, suggesting that while the types of abnormalities are similar, the severity and frequency of occurrence differ. This finding is consistent with Purdue et al., who questioned the necessity of routine monitoring in patients with no initial ECG abnormalities [32]. However, in our study, the severe trauma sustained by train surfers justifies comprehensive cardiac monitoring, as also recommended by Bailey et al. for high-risk cases [33,34].

The significantly higher resuscitation rate among train surfers compared to work-related injuries suggests a greater physiological burden and risk associated with their injuries. The observed disparity in biomarker levels, with creatine kinase (CK) and myoglobin levels markedly higher in train surfers, further supports this finding. Elevated troponin levels in train surfers compared to work-related cases (309.08 ng/L vs. 27.91 ng/L) reflect more extensive myocardial damage, consistent with Robinson’s review, which suggests that electrical injuries can lead to subtle yet significant cardiac dysfunction over time [35].

Our findings support the need for prolonged monitoring, particularly in high-risk groups such as train surfers, given the potential for delayed arrhythmias, as observed in Jensen et al.’s study [36]. The presence of rare findings such as negative T waves and bigeminus with right bundle branch block (RBBB) underscores the necessity of individualized patient management strategies. As recommended by Cunningham and Bailey et al., patients with high-voltage exposure or abnormal initial ECG findings should undergo extended observation [34,37].

When comparing our findings to those of Pilecky et al. and Seyfrydova et al., both of which focused predominantly on low-voltage electrical injuries, marked differences in cardiac outcomes and biomarker profiles become evident [22,38]. In the study by Pilecky et al., involving 480 mostly low-voltage cases, in-hospital and 30-day mortality were zero, and clinically relevant arrhythmias were exceedingly rare [38]. Troponin I and CK-MB elevations were minimal, and routine ECG monitoring was deemed unnecessary. Similarly, Seyfrydova et al. analyzed 333 cases—91.6% of which were low-voltage—and reported only 5.7% troponin elevations, with no instances of delayed malignant arrhythmias [22]. In contrast, our cohort, which included only high-voltage injuries, demonstrated significantly higher rates of cardiac failure (21.6%), resuscitation (27.5%), and ECG abnormalities (55.9%), particularly among train-surfing patients. Moreover, cardiac biomarkers such as troponin, creatine kinase, and myoglobin were markedly elevated, especially in cases involving vertical current flow. These differences likely reflect the distinct pathophysiological burden of high-voltage trauma and reinforce the importance of injury mechanisms and the current direction in clinical risk stratification. While low-voltage injuries may allow for more conservative management, our findings emphasize the necessity for more aggressive monitoring and individualized assessment in high-voltage cases. Together, these comparisons highlight a fundamental gap in the literature: current guidelines often fail to differentiate between low- and high-voltage electrical trauma despite their significantly divergent clinical courses and complication profiles.

Future studies should focus on long-term follow-up to assess the persistence and evolution of ECG abnormalities, as suggested by Guinard et al., who reported persistent myocardial dysfunction months after electrical injuries [31]. Additionally, incorporating advanced imaging modalities such as cardiac MRI or scintigraphy could provide deeper insights into myocardial integrity and perfusion post-injury.

## 5. Conclusions

This study contributes valuable long-term data on high-voltage injuries caused by train surfing, a rare but devastating phenomenon. Unlike previous publications, we included a comparative analysis with work-related electrical injuries and performed a multivariate logistic regression to identify predictors of in-hospital mortality. The analysis revealed that total body surface area (TBSA), the need for resuscitation, and the presence of acute kidney failure were independent predictors of mortality. Specifically, TBSA (OR 1.08, 95% CI 1.02–1.15, *p* = 0.009), resuscitation (OR 4.56, 95% CI 1.11–18.79, *p* = 0.035), and kidney failure (OR 3.27, 95% CI 1.09–9.76, *p* = 0.034) were significantly associated with increased mortality. While membership in the train-surfing group showed a trend toward higher mortality (OR 2.11, *p* = 0.129), this did not reach statistical significance. These findings underscore the multifactorial nature of high-voltage trauma and highlight the severe systemic impact observed in train-surfing incidents. The broad spectrum of injury patterns—ranging from cardiac arrhythmias to secondary traumatic injuries—further emphasizes the need for targeted prevention and early intensive care interventions.

Our results suggest that the direction of electrical current passing through the intrinsic cardiac axis plays a significant role in the observed changes in cardiac biomarkers and rhythm disturbances in this patient cohort. The pathway of electrical conduction through the heart’s conduction system may influence the extent and nature of myocardial involvement, potentially explaining the higher incidence of cardiac complications observed in specific injury patterns.

## Figures and Tables

**Table 1:** Demographic data.

Variable	Train-Surfing	Work-Related	*p*-Value
Total Patients	32	70	
Male	31/32 (97%)	66/70 (94%)	946
Female	1/32 (3%)	4/70 (6%)	
Median Age (years)	19 (13–36)	34 (18–59)	<0.001
TBSA Mean (±SD)	47.6 ± 20.1	25.4 ± 17.8	<0.001
Associated Trauma	18/32 (56.25%)	19/70 (27.14%)	8
Cardiac Failure	12/32 (37.5%)	10/70 (14.29%)	15
Resuscitation Required	14/32 (43.75%)	11/70 (15.71%)	5
Kidney Failure	14/32 (43.75%)	15/70 (21.43%)	31
Dialysis Required	11/32 (34.38%)	11/70 (15.71%)	22

**Table 2:** Definition of current flow types.

Current Flow Type	Definition
Vertical	Entry point is at the head, upper torso, or upper extremities; exit point is in the lower extremities (e.g., hand → foot, head → leg). Current travels top-to-bottom.
Diagonal	Entry and exit points are on opposite quadrants (e.g., right hand → left foot, shoulder → contralateral thigh), crossing the body’s axis obliquely.
Horizontal	Entry and exit points are on the same horizontal plane (e.g., right hand →left hand, arm → chest, foot → foot). Current travels side-to-side or across limbs.
Undefined	No clearly documented entry or exit point, or insufficient clinical data.

**Table 3:** ECG findings.

	Train Surfers	Work Related	Total
No ECG Findings	13.0	34.0	47.0
Sinus Tachycardia	5.0	13.0	18.0
Ventricular Extrasystoles	2.0	4.0	6.0
Atrial Fibrillation	2.0	4.0	6.0
Sinus Bradycardia	2.0	4.0	6.0
Sinus Tachycardia + RBBB	2.0	0.0	2.0
Ventricular Tachycardia	1.0	0.0	1.0
Ventricular Fibrillation	1.0	4.0	5.0
Negative T Waves + RBBB	1.0	0.0	1.0
Negative T Waves	1.0	0.0	1.0
Supraventricular Tachycardia	1.0	2.0	3.0
Sinus Bradycardia + RBBB	1.0	1.0	2.0
ST Elevation	0.0	2.0	2.0
Atrial Fibrillation + ST Elevation	0.0	1.0	1.0
Bigeminus + RBBB	0.0	1.0	1.0
Total Cases	32.0	70.0	102.0

**Table 4:** Predictors of mortality.

Variable	*p*-Value	Interpretation
Total Body Surface Area (TBSA)	0.009	OR 1.08 (95% CI 1.02–1.15); significant
Resuscitation	0.035	OR 4.56 (95% Cl 1.11–18.79); significant
Acute Kidney Failure	0.034	OR 3.27 (95% CI 1.09–9.76); significant
Train-surfing Group	0.129	OR 2.11 (95% CI 0.79–5.63); trend
ECG findings (overall)	0.490	Not significant
Model stability and confounder adjustment		VIF < 2.5 for all variables; multivariate model adjusted for age, ECG findings, kidney injury and group assignment

**Table 5:** Predictors of cardiac failure.

Variable	*p*-Value	Interpretation
Train-surfing	0.158	Trend, not significant
Age	0.968	Not significant
ECG Abnormalities	0.997	Not significant; estimate unstable
Entry/Exit Sites	>0.267	Not significant
Model stability and confounder		Limited convergence due to low event number; no
adjustment		multicollinearity detected

**Table 6:** Predictors of kidney failure.

Variable	*p*-Value	Interpretation
ECG Abnormalities	0.007	Significant predictor
Train-surfing	0.151	Trend, not significant
Age	0.860	Not significant
Entry/Exit Sites	>0.681	Not significant
Model stability and confounder adjustment	-	VIF < 2.5; variables selected based on clinical plausibility and data availability

## Data Availability

The original contributions presented in the study are included in the article, further inquiries can be directed to the corresponding authors.

## References

[1] Korkiamaki A., Kinnunen E., Lindford A., Vuola J. (2024). Electrical burns in train climbers treated in the Helsinki Burn Centre during the last 30 years. Scand. J. Trauma Resusc. Emerg. Med..

[2] Shih J.G., Shahrokhi S., Jeschke M.G. (2017). Review of Adult Electrical Burn Injury Outcomes Worldwide: An Analysis of Low-Voltage vs High-Voltage Electrical Injury. J. Burn Care Res..

[3] Stockly O.R., Wolfe A.E., Espinoza L.F., Simko L.C., Kowalske K., Carrougher G.J., Gibran N., Bamer A.M., Meyer W., Rosenberg M. (2020). The impact of electrical injuries on long-term outcomes: A Burn Model System National Database study. Burns.

[4] Ding H., Huang M., Li D., Lin Y., Qian W. (2020). Epidemiology of electrical burns: A 10-year retrospective analysis of 376 cases at a burn centre in South China. J. Int. Med. Res..

[5] Gille J., Schmidt T., Dragu A., Emich D., Hilbert-Carius P., Kremer T., Raff T., Reichelt B., Siafliakis A., Siemers F. (2018). Electrical injury—A dual center analysis of patient characteristics, therapeutic specifics and outcome predictors. Scand. J. Trauma Resusc. Emerg. Med..

[6] Goyal D., Jagne N., Dhiman A., Patil V., Rattan A. (2021). High voltage electrical injuries: Outcomes & 1-year follow-up from a level 1 trauma centre. Int. J. Burn. Trauma.

[7] Hussmann J., Kucan J.O., Russell R.C., Bradley T., Zamboni W.A. (1995). Electrical injuries--morbidity, outcome and treatment rationale. Burns.

[8] Maghsoudi H., Adyani Y., Ahmadian N. (2007). Electrical and lightning injuries. J. Burn Care Res..

[9] Sternick I., Gomes R.D., Serra M.C., Radwanski H.N., Pitanguy I. (2000). “Train surfers”: Analysis of 23 cases of electrical burns caused by high tension railway overhead cables. Burns.

[10] Koenig V., Tratnig-Frankl P., Pittermann A., Windpassinger M., Joestl J., Aszmann O. (2024). Train Climbing-A new old trend in adolescents: Treatment of high voltage injuries and planning of a pilot project to raise awareness. Wien. Klin. Wochenschr..

[11] Cuculic D., Sosa I. (2019). “Selfie”-related electrocution. Forensic Sci. Med. Pathol..

[12] Koller J. (1991). High-tension electrical-arc-induced thermal burns caused by railway overhead cables. Burns.

[13] Lumenta D.B., Vierhapper M.F., Kamolz L.P., Keck M., Frey M. (2011). Train surfing and other high voltage trauma: Differences in injury-related mechanisms and operative outcomes after fasciotomy, amputation and soft-tissue coverage. Burns.

[14] Khor D., AlQasas T., Galet C., Barrash J., Granchi T., Bertellotti R., Wibbenmeyer L. (2023). Electrical injuries and outcomes: A retrospective review. Burns.

[15] Fordyce T.A., Kelsh M., Lu E.T., Sahl J.D., Yager J.W. (2007). Thermal burn and electrical injuries among electric utility workers, 1995–2004. Burns.

[16] Cancio L.C., Jimenez-Reyna J.F., Barillo D.J., Walker S.C., McManus A.T., Vaughan G.M. (2005). One hundred ninety-five cases of high-voltage electric injury. J. Burn Care Rehabil..

[17] Fish R.M., Geddes L.A. (2009). Conduction of electrical current to and through the human body: A review. Eplasty.

[18] Kym D., Seo D.K., Hur G.Y., Lee J.W. (2015). Epidemiology of electrical injury: Differences between low- and high-voltage electrical injuries during a 7-year study period in South Korea. Scand. J. Surg..

[19] Zack F., Schau H., Dalchow A., Rock M., Blaas V., Buttner A. (2020). Lesions and characteristic injury patterns caused by high-voltage fault arcs. Int. J. Leg. Med..

[20] Sanford A., Gamelli R.L. (2014). Lightning and thermal injuries. Handb. Clin. Neurol..

[21] Gilles F., Nicot F., Boyer C., Georges J.L. (2023). Acute myocardial damage after electrical injury assessed by MRI. BMJ Case Rep..

[22] Seyfrydova M., Rokyta R., Rajdl D., Huml M. (2023). Arrhythmias and laboratory abnormalities after an electrical accident: A single-center, retrospective study of 333 cases. Clin. Res. Cardiol..

[23] Hansen S.M., Riahi S., Hjortshoj S., Mortensen R., Kober L., Sogaard P., Torp-Pedersen C. (2017). Mortality and risk of cardiac complications among immediate survivors of accidental electric shock: A Danish nationwide cohort study. BMJ Open.

[24] Waldmann V., Narayanan K., Combes N., Jost D., Jouven X., Marijon E. (2018). Electrical cardiac injuries: Current concepts and management. Eur. Heart J..

[25] Waldmann V., Narayanan K., Marijon E. (2021). Electrical injury-triggered ventricular arrhythmia in a patient with a pacemaker: Highlighting the importance of cardiac monitoring. Europace.

[26] Thomson A.J. (2013). Physical properties of electricity. J. Minim. Invasive Gynecol..

[27] Zhu Z.X., Xu X.G., Li W.P., Wang D.X., Zhang L.Y., Chen L.Y., Liu T.Y. (2003). Experience of 14 years of emergency reconstruction of electrical injuries. Burns.

[28] Ferreiro I., Melendez J., Regalado J., Bejar F.J., Gabilondo F.J. (1998). Factors influencing the sequelae of high tension electrical injuries. Burns.

[29] Garcia-Sanchez V., Gomez Morell P. (1999). Electric burns: High- and low-tension injuries. Burns.

[30] Handschin A.E., Vetter S., Jung F.J., Guggenheim M., Kunzi W., Giovanoli P. (2009). A case-matched controlled study on high-voltage electrical injuries vs thermal burns. J. Burn Care Res..

[31] Guinard J.P., Chiolero R., Buchser E., Delaloye-Bischof A., Payot M., Grbic A., Krupp S., Freeman J. (1987). Myocardial injury after electrical burns: Short and long term study. Scand. J. Plast. Reconstr. Surg..

[32] Purdue G.F., Hunt J.L. (1986). Electrocardiographic monitoring after electrical injury: Necessity or luxury. J. Trauma..

[33] Bailey B., Gaudreault P., Thivierge R.L. (2000). Experience with guidelines for cardiac monitoring after electrical injury in children. Am. J. Emerg. Med..

[34] Bailey B., Gaudreault P., Thivierge R.L. (2007). Cardiac monitoring of high-risk patients after an electrical injury: A prospective multicentre study. Emerg. Med. J..

[35] Robinson N.M., Chamberlain D.A. (1996). Electrical injury to the heart may cause long-term damage to conducting tissue: A hypothesis and review of the literature. Int. J. Cardiol..

[36] Jensen P.J., Thomsen P.E., Bagger J.P., Norgaard A., Baandrup U. (1987). Electrical injury causing ventricular arrhythmias. Br. Heart J..

[37] Cunningham P.A. (1991). The need for cardiac monitoring after electrical injury. Med. J. Aust..

[38] Pilecky D., Vamos M., Bogyi P., Muk B., Stauder D., Racz H., Nyolczas N., Duray G.Z., Zacher G., Zima E. (2019). Risk of cardiac arrhythmias after electrical accident: A single-center study of 480 patients. Clin. Res. Cardiol..

